# The DAWGPAWS pipeline for the annotation of genes and transposable elements in plant genomes

**DOI:** 10.1186/1746-4811-5-8

**Published:** 2009-06-19

**Authors:** James C Estill, Jeffrey L Bennetzen

**Affiliations:** 1 Department of Plant Biology, The University of Georgia, Athens, Georgia 30602-7271, USA; 2 Department of Genetics, The University of Georgia, Athens, Georgia 30602-7223, USA

## Abstract

**Background:**

High quality annotation of the genes and transposable elements in complex genomes requires a human-curated integration of multiple sources of computational evidence. These evidences include results from a diversity of *ab initio *prediction programs as well as homology-based searches. Most of these programs operate on a single contiguous sequence at a time, and the results are generated in a diverse array of readable formats that must be translated to a standardized file format. These translated results must then be concatenated into a single source, and then presented in an integrated form for human curation.

**Results:**

We have designed, implemented, and assessed a Perl-based workflow named DAWGPAWS for the generation of computational results for human curation of the genes and transposable elements in plant genomes. The use of DAWGPAWS was found to accelerate annotation of 80–200 kb wheat DNA inserts in bacterial artificial chromosome (BAC) vectors by approximately twenty-fold and to also significantly improve the quality of the annotation in terms of completeness and accuracy.

**Conclusion:**

The DAWGPAWS genome annotation pipeline fills an important need in the annotation of plant genomes by generating computational evidences in a high throughput manner, translating these results to a common file format, and facilitating the human curation of these computational results. We have verified the value of DAWGPAWS by using this pipeline to annotate the genes and transposable elements in 220 BAC insertions from the hexaploid wheat genome (*Triticum aestivum *L.). DAWGPAWS can be applied to annotation efforts in other plant genomes with minor modifications of program-specific configuration files, and the modular design of the workflow facilitates integration into existing pipelines.

## Background

Genomic sequence assemblies are rapidly being published for a great number of species [1,2]. The sequence data used to produce genome assemblies are being generated at ever-increasing rates for reduced costs [3], indicating that the genomes of many more plant species will be *de novo *sequenced in coming years. The relative value of these sequencing efforts is a direct function of the accuracy of the annotation of the resultant sequence assemblies. Genome annotation seeks to delineate the sequence features that occur on the genome, thereby permitting definition of the biological processes responsible for these features [4]. In plants, the sequence characteristics that are most critical to our interpretation of gene function and genome evolution include both genes and transposable elements (TEs) [5,6].

Identification of the genes that have been uncovered in assembled genome sequence data can utilize evidence from both *ab initio *gene annotation programs as well as sequence similarity searches against databases of previously identified proteins and expressed RNA [4,7,8]. The *ab initio *gene finding programs derive full gene models from DNA sequence data based solely on knowledge of the sequence features associated with protein coding domains. Sequence alignments can refine the exon-intron boundaries of these models and provide evidence that computationally predicted genes are actually transcribed *in vivo*. Existing software can automatically synthesize these data to derive combined evidence gene models [9,10].

While this combination of *ab initio *and homology-based approaches have been used to accurately annotate genes in a number of eukaryotic genomes, plant genome annotation efforts cannot focus solely on the annotation of genes due to the risk of conflating genes with transposable elements [11]. Many TEs contain open reading frames (ORFs) that generate the proteins required for TE transposition. The *ab initio *gene annotation programs will often annotate these TE ORFs as genes. Since most TE genes are expressed and represented in cDNA libraries, homology-based searches will indicate that these ORFs are transcribed and they thus may be considered legitimate gene predictions. Simply removing the high-copy-number candidate genes does not alleviate this problem because some true gene families are highly abundant while not all transposable elements are highly repetitive [12]. These erroneous gene annotations are especially problematic in plant genomes where transposable elements make up the majority of sequenced genome space. Since these false positive gene predictions cannot be mitigated by gene prediction methods alone, plant genome annotation must directly annotate TEs in order to remove them from the gene candidate list.

Similar to the prediction of genes, accurate identification of the TEs in genomic sequence data combines homology-based searches and *ab initio *results [13-15]. Tools for *ab initio *transposable element discovery can exploit the fact that many families of TEs occur in high copy number within a host genome [16-18], or they can utilize diagnostic structural features such as tandem inverted repeats (TIRs) or long terminal repeats (LTRs) that delineate an individual TE insertion [19-21]. Homology-based searches of transposable elements are facilitated by specialized tools [22-25] that make use of databases of previously identified TEs [26-29] or leverage repetitive data from the sequenced genome [30-32].

The gold standard of genome annotation is the integration and curation of multiple computational results by a knowledgeable biologist [11]. This approach has been advocated for the structural annotation of genes [4,11], as well as transposable elements [33]. A limitation of the manually-curated multiple-evidences approach is that the process requires the combination of computational results from a disparate set of independent annotation programs. The output of this software has been designed to maximize readability by humans and not to facilitate integration of results across programs. Furthermore, these tools are often designed to work on a single contiguous sequence (contig) at a time, while many annotation efforts require the generation of computational results for thousands of assembled contigs. Computational workflow suites that seek to aid in plant genome annotation must therefore overcome these limitations while facilitating the human interpretation of the computational results contributing to a biological annotation.

Here, we introduce an annotation suite that allows for computational evidences to be generated in an automated fashion, integrates the results from multiple programs and facilitates the human curation of these computational results. This suite was designed to assist a Distributed Annotation Working Group (DAWG) approach for a Pipeline to Annotate Wheat Sequences (PAWS), and we hereafter refer to this effort as DAWGPAWS.

## Implementation

**Figure 1:**
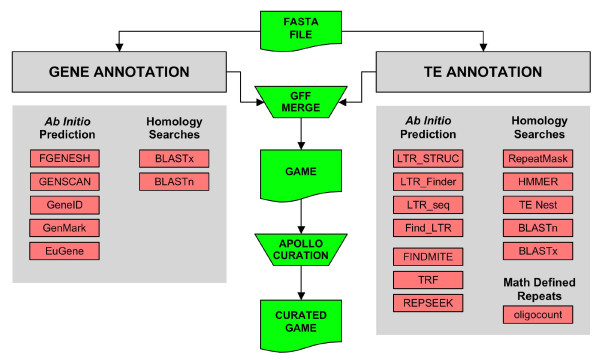
**An overview of the workflow supported by the current version of the DAWGPAWS suite of programs**.

The DAWGPAWS workflow (Figure 1) is distributed as a suite of individual command line interface (CLI) programs written in the Perl programming language. Generally, each program is tailored for an individual step in the annotation process, and it can be used independently of all other programs in the package. This allows users to design an individualized annotation pipeline by selecting those computational components that are most appropriate to their annotation efforts. This modular design also facilitates using DAWGPAWS in a high throughput cluster-computing framework. Large-scale annotation jobs can be split across compute nodes by contigs being annotated as well as by the computational process used to generate computational results.

A common thread to each component of the DAWGPAWS package is that computational evidences are translated from the native annotation program output into the standard general feature format (GFF) [34]. The GFF file format facilitates integration of multiple computational results. This format can be directly curated by any biologist using standard sequence curation and visualization tools such as Apollo [35], Artemis [36], GBrowse [37], the UCSC genome browser [38] or the Ensembl Genome Browser [39]. The GFF files also provide a standard format for loading annotation results to relational database schemas such as BioSQL [40] or CHADO [41].

One of the main sets of scripts in the DAWGPAWS package is the batch run program set (Table 1). All of these scripts are designed to run individual annotation programs in a high throughput batch mode. They take as their input a directory of sequence files that are to be annotated and a configuration file describing the sets of parameters to use for each sequence file. The output of these batch scripts includes the original output from the annotation program as well as this output translated to the GFF format. The resulting files are stored in a predefined directory structure that allows users to quickly locate the original annotation results as well as the GFF copy. These batch programs exist for both gene and TE annotation results. The *ab initio *gene annotation programs supported by these scripts include EuGène [9], GeneID [42], GeneMark.hmm [43], and Genscan [44]. The *ab initio *TE annotation programs that can be run in batch mode are Find_LTR [45], LTR_STRUC [20], LTR_FINDER [21], LTR_seq [46], FINDMITE [19], and Tandem Repeats Finder [47]. Batch mode scripts also support TE annotation using HMMER [48], NCBI-BLAST [49], RepeatMasker [22], and TEnest [24]. The full set of gene and TE annotation programs that can be run in batch mode are summarized in Table 1.

**Table 1:** DAWGPAWS annotation scripts for generating computational annotation results in batch mode.

**Annotation Program**	**Result Type**	**DAWGPAWS Script**
EuGène [9]	Gene *ab initio *and automated combined evidence	batch_eugene.pl

GeneID [42]	Gene *ab initio*	batch_geneid.pl

GeneMark.hmm [43]	Gene *ab initio*	batch_genemark.pl

Genscan [44]	Gene *ab initio*	batch_genescan.pl

Find_LTR [45]	TE *ab initio*	batch_findltr.pl*

LTR_STRUC [20]	TE *ab initio*	batch_ltrstruc.vbs

LTR_FINDER [21]	TE *ab initio*	batch_ltrfinder.pl*

LTR_seq [46]	TE *ab initio*	batch_ltrseq.pl*

FINDMITE [19]	TE *ab initio*	batch_findmite.pl*

Tandem Repeats Finder [47]	Repeat *ab initio*	batch_trf.pl

HMMER [48]	TE homology	batch_hmmer.pl*

NCBI-BLAST [49]	TE and gene homology	batch_blast.pl*

RepeatMasker [22]	TE homology	batch_repmask.pl*

TEnest [24]	TE homology	batch_tenest.pl

These scripts operate on a directory of FASTA files, and generate the native results of the annotation program as well as the GFF file format. The exception is the batch_ltrstruc.vbs visual basic script that must be used in conjunction with cnv_ltrstruc2gff.pl to generate results in GFF.

* Indicates programs that make use of a configuration file. The nature and format of the configuration file for these programs is described in the individual help file for those programs.

In addition to the batch run programs, scripts that convert an individual annotation program output to GFF are also available (Table 2). These programs allow an existing annotation result to be specified, or they can take advantage of UNIX standard streams. If an input file is not specified, the conversion scripts will expect input from the standard input stream. Likewise, if the output path is not specified, these programs will write the output to a standard output stream. Accepting standard input and output streams facilitates using these programs as supplements to an existing workflow. For example, data can be piped directly from the output stream of an annotation program to a DAWGPAWS converter, and then piped on to a parser that loads the GFF formatted result to a database. These conversion programs provide the ability to support conversion of output from programs such as FGENESH [50,51] and RepSeek [52] that are not supported by batch scripts in DAWGPAWS.

**Table 2:** DAWGPAWS scripts for conversion of annotation results from native program output to GFF.

**Annotation Program**	**Result Type**	**DAWGPAWS Script**
FGENESH [50,51]	Gene *ab initio*	cnv_fgenesh2gff.pl

GeneMark.hmm [43]	Gene *ab initio*	cnv_genemark2gff.pl

Find_LTR [45]	TE *ab initio*	cnv_findltr2gff.pl

LTR_FINDER [21]	TE *ab initio*	cnv_ltrfinder2gff.pl

LTR_seq [46]	TE *ab initio*	cnv_ltrseq2gff.pl

LTR_STRUC [20]	TE *ab initio*	cnv_ltrstruc2gff.pl

RepSeek [52]	TE *ab initio*	cnv_repseek2gff.pl

NCBI-BLAST [49]	TE and gene homology	cnv_blast2gff.pl

RepeatMasker [22]	TE homology	cnv_repmask2gff.pl

TEnest [24]	TE homology	cnv_tenest2gff.pl

The DAWGPAWS suite also includes specialized tools for TE annotation. For identification of the highly repetitive regions of a contig, the seq_oligocount.pl program can count the occurrence of oligomers in the query sequence against an index of random shotgun sequences. This program generates all oligomers of length k from the query sequence, and uses the vmatch program [53] to determine the number of these k-mers that occur in a random shotgun sequence data set generated by mkvtree [53]. The output of this program is a GFF file indicating the count of these k-mers in the shotgun sequence dataset. These results may be used to identify the mathematically defined repeats in the query sequence, as well as provides a means to visualize low-copy-number runs in the query sequence [54].

In addition to the gene and TE annotation-specific scripts included in the DAWGPAWS package, helper applications are also included (Table 3). These CLI programs fulfill needs that occur when generating annotation results. They allow for file conversion such as the conversion of GFF to game.xml format or the conversion of a lowercase masked sequence file to a hard masked sequence file. They also prepare the sequence files for annotation by shortening FASTA headers as required by some programs, or by splitting a single FASTA file containing multiple records into multiple FASTA files containing single record files. The ability to generate Euler Diagrams is also supported via the vennseq.pl conversion script that formats GFF file data for input into the VennMaster program [55].

**Table 3:** Additional helper scripts included in the DAWGPAWS package.

**DAWGPAWS Script**	**Purpose**
cnv_gff2game.pl	Converts GFF files to the game.xml format.

cnv_game2gff3.pl	Converts game.xml files to the GFF3 format.

batch_hardmask.pl	Given a directory of lowercase masked sequence files, this will replace lowercase residues with an N or X to indicate masking.

dir_merge.pl	Given annotation results scattered across multiple directories, this program can merge the results into subdirectories in a single parent directory.

vennseq.pl	Given GFF annotation results from multiple methods, this program generates a Euler Diagram of these features using the VennMaster program [55]

batch_findgaps.pl	This program will annotate gaps in the query sequences in the input directory.

clust_write_shell.pl	This program writes shell scripts to run DAWGPAWS in a cluster environment running the Platform LSF queuing system.

cnv_seq2dir.pl	Given a FASTA file with multiple sequence files, this program generates a separate FASTA file for each sequence record. The sequence files produced are named using the sequence ID in the FASTA header in the input file.

fasta_merge.pl	This program merges all FASTA files in a directory into a single FASTA file.

fasta_shorten.pl	This program shortens the FASTA header by limiting the header length, or splitting the header by a delimiting character. Some annotation programs are limited by the length of the FASTA header that is accepted, and this programs allows input files to meet this limitation.

fetch_tenest.pl	Fetches multiple results from the Plant GDB TEnest server and converts the results to GFF.

gff_seg.pl	Given a GFF file that contains point or segment data, this will extract segments with score values that exceed a threshold value.

ltrstruc_prep.pl	Because the LTR_STRUC program only runs under the windows environment, this program converts FASTA sequences in UNIX to DOS line endings and generates the files name and flist file required for LTR_STRUC.

seq_oligiocount.pl	This program allows for the generation of a GFF file that counts the number of times an oligomer in the genomic contig occurs in a reference shotgun sequence database.

A CLI interface was selected for DAWGPAWS to facilitate the use of our applications in a cluster-computing environment, and to provide stability in program interface across multiple operating systems. While command line interface programs may be daunting to some users, every effort has been made to simplify their use. All of the CLI programs included in the DAWGPAWS suite follow consistent protocols for command line options (Table 4). Help files or full program manuals are available from the command line within all programs by invoking the – help or – man options. These application manuals are also available in HTML form on the DAWGPAWS website along with a general program manual describing the installation and use of a local implementation of the DAWGPAWS package [56]. This documentation is also included in the downloadable release of DAWGPAWS.

**Table 4:** Common command line options used throughout the DAWGPAWS suite of programs.

**Option**	**Description**
--indir *or* --infile	For batch scripts, this indicates the input directory containing the FASTA files to annotate. For conversion scripts, this indicates the input file to convert from the native format to the GFF format.

--outdir *or* --outfile	For batch scripts, this indicates the output directory containing the annotation results for the program and the GFF results. For conversion scripts, this indicates the path to the GFF output file.

--config	For programs that make use of a configuration file, this indicates the path to the configuration file to use.

--seqname	For conversion scripts, this indicates the sequence id to use in the GFF output file.

--param	For conversion scripts, this indicates the name of that parameter set used with the annotation program. This option allows the user to distinguish among multiple parameter sets for the same annotation program, and this parameter name is appended to the source column of the GFF output file.

--program	For conversion scripts, this indicates the name of the program used to generate the annotation result.

--version	Print the current version of the script.

--usage	Print a short program usage message.

--help	Print a short help message including the common usage and all program options available at the command line.

--man	Print the full program manual.

--verbose	This will run the program with maximum verbosity. This option will generate status updates while the program is running, and will maximize the error reporting functions of the script. All verbose statements are written to the standard error output stream.

## Results and discussion

**Figure 2:**
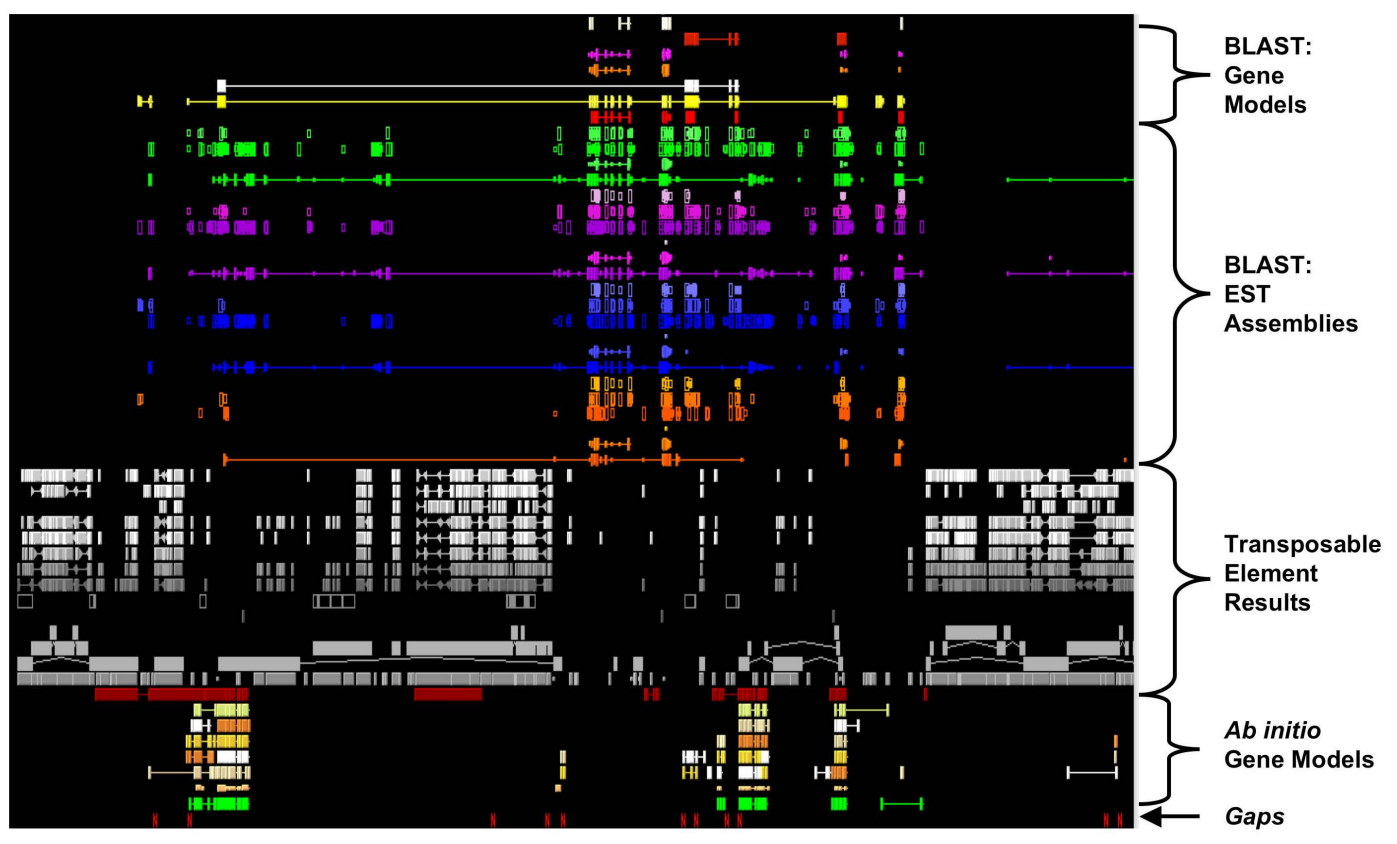
**Screen capture image of gene and TE annotation results visualized in the Apollo genome annotation program**. This example shown is for a wheat BAC that has been annotated and curated with the assistance of DAWGPAWS.

The computational annotation results generated by DAWGPAWS can be directly imported into any genome annotation program that supports GFF. We have used the Apollo program [35] to visualize and curate our results for genes and transposable elements in the wheat genome (Figure 2). Since the game xml file format is the most stable way to store annotation results in Apollo, it is generally useful to first convert GFF files to the game xml format before beginning curation of computational results. The visual display of computational results in Apollo is modified by a tiers configuration file. This file controls how and where individual computational and annotation results are drawn on the annotation pane. The tiers file used in these annotation efforts is included in the DAWGPAWS download package, and it can serve as a starting point for generating individualized tier files for other plant annotation efforts. As an alternative to Apollo, it is also possible to curate computational results using the Artemis sequence visualization program [36].

**Figure 3:**
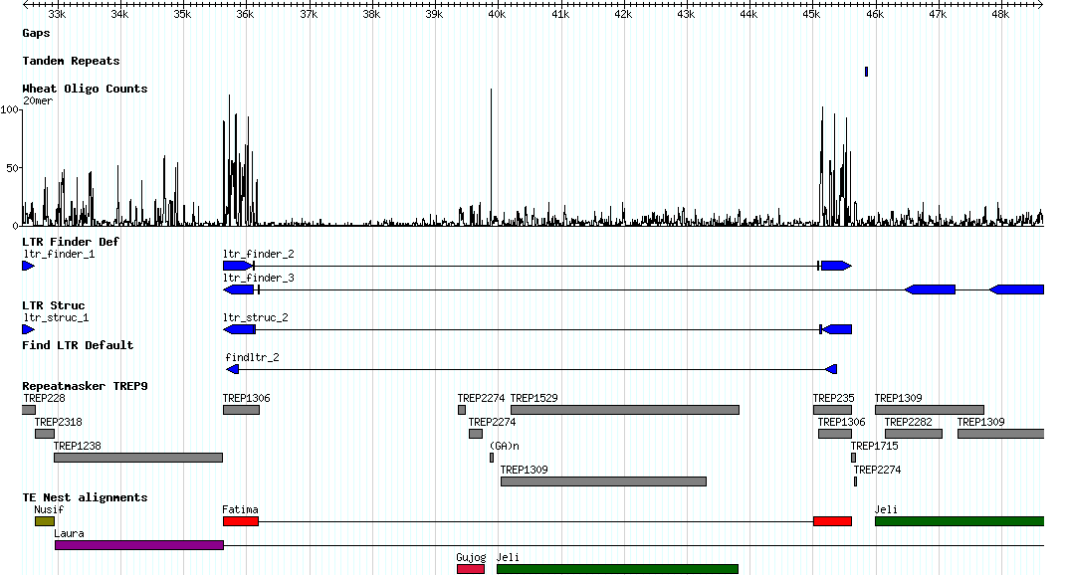
**Screen capture image of the TE annotation results and oligomer counts visualized in the GBrowse genome annotation visualization program**. The example shown is for a 15 kb segment of a BAC with a wheat DNA insert.

The GBrowse package [37] can also visualize GFF formatted annotations, and has proven to be a useful method for visualizing TE results. GBrowse makes use of core images called glyphs that are used to draw sequence features along a genome. The available glyphs in GBrowse can be supplemented by writing additional Perl modules, and we have generated TE glyphs that allow visualization of the biologically relevant features of TEs. GBrowse also has the capability to draw histograms along the sequence contigs. GBrowse can thus combine TE glyphs and histograms to provide an informative visualization of the distribution of mathematically defined repeats and the structural features of TEs (Figure 3). The current drawback to visualizations in GBrowse is that the program is intended to serve as a static visualization tool, and does not provide the means for the curation and combination of computational results. It would therefore be helpful if the current curation programs for gene annotation, such as Apollo or Artemis, directly addressed the needs of TE annotation curation and developed glyphs for the major classes of TEs.

In addition to visualization and curation of the annotated DNA, it is also possible to transfer the DAWGPAWS results into existing database schema. For example, the CHADO database [41] can make use of the gmod_bulk_load_gff3.pl program [57] that can load GFF3 format files into a CHADO database. In the DAWGPAWS package, the GFF3 format files from curated results can be generated with the cnv_game2gff3.pl program. These curated results could then be stored in a local implementation of the CHADO database. The BioSQL database schema [40] also includes a bp_load_gff.pl script that can load GFF results into the database schema.

The DAWGPAWS annotation framework has a number of features that make it a good choice to facilitate the workflow in plant genome annotation. The use of configuration files makes it fairly easy to modify the annotation workflow for the species of interest. The configuration files also makes it quite easy to generate results with multiple parameter sets for an individual program. Using multiple parameter sets will be especially useful when working with a genome that has not been annotated before, and for which appropriate annotation parameters have not been identified. Also, while previous annotation pipelines have focused on gene annotation, the DAWGPAWS suite maximizes the quality of TE annotation results. Most plants contain genomes with sizes > 5000 Mb [58], and are therefore expected to contain more than 80% TEs [59], so efficiently dealing with this large number and diverse set of mobile DNAs is necessary for effective genome annotation.

The current focus of DAWGPAWS in our laboratory is the structural annotation of the genes and TEs in a genome using methods and applications tuned to the Triticeae. In annotation of 220 BACs from hexaploid bread wheat, we found that the DAWGPAWS pipeline increased the rate of individual BAC annotations by twenty-fold. Due to the time required to manually generate annotation results, this previous annotation effort was limited to using the FGENESH annotation program combined with a BLAST search of predicted models against known transposable elements and protein databases [60]. Using this method, annotators could annotate a single BAC in one to two days. The implementation of the DAWGPAWS pipeline increased the speed of annotation to ten-fifteen BACs per person per day. Furthermore, the quality of both TE and gene prediction were also seen to improve with the use of DAWGPAWS. This was due, at least in part, to the larger number of complementary programs for TE and gene discovery that could be conveniently employed in each BAC annotation. Specifically, the inclusion of *ab initio *TE prediction programs allowed for the identification of new families of LTR retrotransposons that would have been missed in our previous annotation efforts. Predicted gene models that span these newly discovered families would not have been identified as TEs in the exclusively homology-based searches that were previously used.

Future development of DAWGPAWS will incorporate tools for the functional annotation of the predicted genes. Currently, functional annotation can be done within the Apollo program by manually selecting individual gene models and BLASTing these results against appropriate databases. A batch run support for additional local alignment search tools will also be added. The use of NCBI-BLAST is sufficient for most comparisons of sequence contigs against reference databases, but programs such as BLAT [61] or sim4 [62] are designed specifically to align ESTs and flcDNAs against assembled genomes. While output from these local alignment tools can be converted to GFF using the existing cnv_blast2gff.pl program in DAWGPAWS, it would be useful to use these packages in a batch run framework similar to the batch_blast.pl program.

Support for additional *ab initio *gene annotation programs will also be added to future releases of DAWGPAWS. Augustus [63] is an *ab initio *annotation program that will be useful for gene annotation that seeks to identify all transcripts derived from a single locus. Support for GENEZILLA [64] and GlimmerHMM [64] gene annotation packages will also be added to future releases of DAWGPAWS. The SNAP program [65] will be added to support the annotation of genomes that have been sequenced *de novo *and lack species-specific HMM model parameterizations. The addition of the PASA [66] program would assist in the annotation of genomes that have large transcript databases that can assist genome annotation. As additional fully-sequenced genomes are added to the plant genomics literature, we can make use of syntenic comparisons and multiple alignments to aid in gene annotation [67] as well as TE annotation [68]. Future development of DAWGPAWS will incorporate syntenic alignment and prediction programs such as SGP2 [69], SLAM [70], and TWINSCAN [71] as they become increasingly relevant to plant genome annotation.

## Conclusion

The DAWGPAWS annotation workflow provides a suite of command line interface programs that can generate computational evidences for human curation in a high-throughput fashion. We have used the DAWGPAWS pipeline to annotate 220 randomly selected BACs with wheat DNA inserts for both gene and TE content. Our curation efforts on the DAWGPAWS output are implemented in the Apollo program. The tiers file used for visualization of this curation are available as part of the DAWGPAWS package.

DAWGPAWS represents an efficient tool for genome annotation in the Triticeae, and can be used in its current form to generate gene and TE computational results for other grass genomes. Minor modifications to the configuration files used by DAWGPAWS can make this program suitable to the generation of computational annotation results for any plant genome. The TE annotation capabilities of DAWGPAWS exceeds any other current genome annotation suite, and makes this package particularly valuable for the great majority of plant genomes, such as wheat or maize, that contain a diverse arrays of TEs that comprise the majority of the nuclear genome.

The DAWGPAWS program has been specifically designed to facilitate use of individual component scripts outside of the entire package. Each script can function independently of all other applications in the package, and programs make use of standard input and standard output streams when possible to facilitate integration into existing pipelines. Since this package is being released under the open source GPL (version 3), the suite and its individual components can be used and modified under the terms of the GPL. Template batch run and conversion scripts are provided in a boilerplate format to facilitate extending DAWGPAWS to additional annotation tools. Furthermore, since we have selected the Perl language for the implementation of our package, the addition of new annotation tools can leverage existing modules in the BioPerl toolkit [72]. These modules include parsers for computational tools useful for predicting alternative splicing [62,61] as well as interfaces for transfer RNA prediction [73]. We also formally invite collaboration in the development of additional DAWGPAWS applications under the auspices of the GNU GPL, as facilitated by the SourceForge subversion repository of the DAWGPAWS source code. Interested collaborators may contact the authors or become member developers of the DAWGPAWS SourceForge project [74].

## Availability and requirements

Project Name: DAWGPAWS Plant Genome Annotation Pipeline

Project Home Page: 

Operating System: Platform Independent

Programming Language: Perl

Other Requirements: BioPerl 1.4, as well as the annotation programs that scripts are dependent upon.

License: GNU General Public License 3

Any restrictions to use by non-academics: No restrictions

## Abbreviations

BAC: Bacterial Artificial Chromosome; cDNA: complementary DNA; CLI: Command Line Interface; EST: Expressed Sequence Tag; flcDNA: full-length complementary DNA; GFF: General Feature Format; GPL: General Public License; HMM: Hidden Markov Model; LTR: Long Terminal Repeat; ORF: Open Reading Frame; pHMM: Profile Hidden Markov Model; TE: Transposable Element

## Competing interests

The authors declare that they have no competing interests.

## Authors' contributions

JE developed the pipeline, wrote the software, and drafted the manuscript. JB conceived the study, oversaw pipeline development, and helped draft the manuscript. All authors read and approved the final manuscript.
